# Cognitive performance from childhood to old age and intergenerational correlations in the multigenerational Young Finns Study

**DOI:** 10.1007/s00415-024-12693-7

**Published:** 2024-09-22

**Authors:** Marja A. Heiskanen, Jaakko Nevalainen, Katja Pahkala, Markus Juonala, Nina Hutri, Mika Kähönen, Eero Jokinen, Tomi P. Laitinen, Päivi Tossavainen, Leena Taittonen, Jorma S. A. Viikari, Olli T. Raitakari, Suvi P. Rovio

**Affiliations:** 1 Research Centre of Applied and Preventive Cardiovascular Medicine, University of Turku, Turku, Finland; 2 Centre for Population Health Research, University of Turku and Turku University Hospital, Turku, Finland; 3 Unit of Health Sciences, Tampere University, Tampere, Finland; 4 Paavo Nurmi Centre, Unit for Health and Physical Activity, University of Turku, Turku, Finland; 5 Department of Medicine, University of Turku and Division of Medicine, Turku University Hospital, Turku, Finland; 6 Tampere Centre for Skills Training and Simulation, Tampere University, Tampere, Finland; 7 Department of Clinical Physiology, Tampere University Hospital and Faculty of Medicine and Health Technology, Tampere University, Tampere, Finland; 8 Department of Pediatric Cardiology, Hospital for Children and Adolescents, Helsinki University Hospital and University of Helsinki, Helsinki, Finland; 9 Department of Clinical Physiology, University of Eastern Finland and Kuopio University Hospital, Kuopio, Finland; 10 Department of Pediatrics, Research Unit of Clinical Medicine, MRC Oulu, University of Oulu, Oulu, Finland; 11 Department of Children and Adolescents, Oulu University Hospital, Oulu, Finland; 12 Department of Pediatrics, Tampere University Hospital, Tampere, Finland; 13 Department of Clinical Physiology and Nuclear Medicine, Turku University Hospital, Turku, Finland; 14 Department of Public Health, University of Turku and Turku University Hospital, Turku, Finland

**Keywords:** Cognitive performance, Lifespan, CANTAB, Neurocognitive testing, Multigenerational study

## Abstract

**Background:**

Cognitive performance changes during the lifespan, but the information is gathered from studies on separate age cohorts. Computerized neurocognitive testing enables efficient and similar assessments for all ages. We investigated (i) the effect of age at different stages of life and (ii) intergenerational correlations across cognitive domains in the multigenerational Young Finns Study.

**Methods:**

Participants in three familiarly related generations (*n* = 6486, aged 7–92 years) performed the Cambridge Neuropsychological Test Automated Battery (CANTAB). Overall cognitive performance and domains representing learning and memory, working memory, information processing, and reaction time were extracted by common principal component analysis from the cognitive data with several age groups. Linear models were used to study the association of age, sex, and education with overall cognitive performance and in the cognitive domains. Age-adjusted intergenerational correlations were calculated.

**Results:**

Learning and memory peaked earlier during the lifespan compared to working memory and information processing, and the rate of decline toward old age differed by domain. Weak intergenerational correlations existed between two consecutive generations but were nonsignificant between grandparents and grandchildren. There was no systematic sex-specific transmission in any cognitive domain.

**Conclusion:**

This study describes the natural course of cognitive performance across the lifespan and proves that cognitive performance changes differently across cognitive domains with weak intergenerational transmission.

**Supplementary Information:**

The online version contains supplementary material available at 10.1007/s00415-024-12693-7.

## Introduction

Cognitive performance develops rapidly from infancy throughout childhood, with differing trajectories between cognitive domains; for example, executive function develops later than memory [1–5]. Young adulthood is considered the peak of cognitive ability [6, 7], whereas in adulthood, cognitive performance starts to decrease with accelerated deterioration in old age [7–9]. Understanding the natural course of the development and decline of cognitive performance in different stages of life is essential to distinguish a cognitive function trajectory that deviates from a healthy one. However, information on cognitive performance across the lifespan is gathered from studies on separate age cohorts using different cognitive tests, whereas large-scale studies assessing cognitive performance using similar testing methodologies for all ages are scarce. In one such study, Swagerman et al. reported nonlinear associations with age in different cognitive domains in a Dutch population ranging in age from 10 to 86 years; however, even in this study, the majority of the participants were adolescent twins [10]. There are no prior large-scale cohort studies in which data on cognitive performance have been systematically collected using identical methods for participants whose age range covers the whole lifespan from childhood to old age in three familiarly related generations.

The heritability of cognitive performance is well established by twin and adoption studies, which have shown that cognitive performance is inherited both genetically and due to environmental influences, such as rearing parents’ educational level [10–12]. The genetic heritability of cognitive performance has recently been assessed by examining the genetic influence of common variants in unrelated individuals [11, 13]. The magnitude of the genetic effect on cognitive performance varies with age, and heritability of general cognitive ability has been suggested to increase from approximately 20% in infancy to 60% in adulthood but to decrease toward old age [12–15]. In addition, heritability may differ by cognitive domain [10, 16]. Previous family studies, mostly of smaller cohorts, have suggested that there is a link between parents’ and offspring’s cognitive performance, that the transmission of cognitive abilities may be sex-specific [17–19] and that it may extend even between grandparents and grandchildren [20]. However, cohorts that could couple sex-specific detailed data on cognitive performance between two generations, and especially between three generations, do not exist.

We leveraged our unique three-generational cohort of the Cardiovascular Risk in Young Finns Study (YFS), which included 6486 participants aged 7 to 92 years, to study (i) the natural course of cognitive performance from childhood to old age and (ii) the intergenerational correlation of cognitive performance across different cognitive domains. This setting enables us to investigate cognitive performance throughout the lifespan with a similar, modern cognitive testing methodology for all participants in our large-scale cohort. Furthermore, exploring the heritability of cognitive abilities across three familiarly related generations provides complementary evidence for previous results derived from twin and adoption studies.

## Materials and methods

### Experimental design

The long-standing national multicenter Cardiovascular Risk in Young Finns Study (YFS) was originally designed to provide evidence on the importance and timing of early-life genetic and environmental exposures in the development of cardiovascular diseases [21]. In 1980, 3596 participants (boys and girls) aged 3–18 years were recruited from five cities and their surrounding rural communities. The original cohort G1 was followed up every 3–6 years. The latest follow-up study was conducted in 2018–2020, in which the data collection was expanded to three familiarly related generations: grandparents (G0; aged 59–92 years), parents (the original YFS participants G1; aged 41–56 years), and offspring (G2; aged 3–37 years). In total, *N* = 12,853 subjects were invited, and *N* = 7341 (57.1%) provided data in the first multigenerational YFS field study. Individuals with diagnosed cognitive impairment and/or severe restrictions in movement abilities were excluded from the study. A total of 6753 (52.5%) participants attended the study visit, while 588 participants provided questionnaire data only. The generation-specific participation rates for participants who attended the study visit were 64.2% for the original participants (G1; *n* = 2064; females 54.8%), 54.5% for the parents of the original participants (G0; *n* = 2146; females 60.6%), and 44.6% for the offspring of the original participants (G2; *n* = 2543; females 54.9%).

### Cognitive performance measurement

Cognitive performance was assessed during the clinical study visit. Due to the blood samples included in the study protocol, the participants arrived at the study visit after fasting and having avoided smoking for at least 4 h. They were also instructed to avoid heavy physical activity and drinking alcohol beginning the previous evening before the study visit. Before the cognitive testing, the subjects were provided with a light snack containing a whole meal oat-based snack biscuit, a fruit/berry oat drink, or weak fruit/berry juice. In total, *N* = 6610 (98%) of the participants who came to the clinical study visit also provided data on cognitive performance. Since the cognitive test battery was modified for 3–6-year-old children, they were excluded from the present study. Hence, the generation-specific numbers of participants who successfully went through the cognitive testing protocol reported in this study were *N* = 2030 (95%) for the original YFS participants (G1), *N* = 2025 (83%) for their parents (G0) and *N* = 2431 (99%) for their children (G2).

Cognitive testing was performed using a computer-based cognitive test battery (CANTAB^®^). The CANTAB^®^ is a computerized, predominantly nonlinguistic and culturally neutral test focusing on a wide range of cognitive domains. The test was performed using a validated touchscreen computer system. The full test battery includes more than 20 individual tests, from which a suitable test battery for each particular study may be selected. The test battery selected for the extended YFS field study included five separate tests that are sensitive enough to capture variation even within the cognitively healthy cohort. The tests selected for the YFS test battery measure four cognitive domains: (1) visual and episodic memory and visuospatial associative learning, (2) short-term and spatial working memory and problem solving, (3) reaction and movement speed and accuracy, and (4) visual processing, recognition, and sustained attention. The test battery was identical for all participants. A study nurse administered the test to all participants and ensured that there was no misunderstanding related to performing the test. Voice-over instructions were provided by the CANTAB^®^ software in Finnish.

First, the participants completed the motor screening (MOT) test, which measures psychomotor speed and accuracy. In the YFS cognitive testing protocol, the MOT test was considered a training procedure that introduced the test equipment to the participants. Simultaneously, the MOT test was used as a screening tool to indicate any difficulties in vision, movement, comprehension, or ability to follow the test instructions. During the MOT test, a series of red crosses were shown in different locations on the screen, and the participants were advised to touch, as quickly as possible, the center of the cross every time it appeared. The MOT test was identical for all participants regardless of age. After the MOT test, four separate tests each measuring a specific cognitive domain were conducted.

***Learning and memory*** was assessed using paired associates learning (PAL) test, which measures visual and episodic memory and visuospatial associative learning and includes aspects of both delayed response procedures and conditional learning. Either 2, 4, 6, 8, or 12 patterns were displayed sequentially in boxes placed on the screen during the PAL test. After that, the patterns were presented in the center of the screen, and the participants were supposed to point to the box in which the particular pattern was previously seen. The test moves on to the next stage if all the patterns are placed in the right boxes. In the case of an incorrect response, all the patterns were redisplayed in their original locations, and another recall phase was performed. The test terminated if the patterns were still incorrectly placed after 4 presentation and recall phases.

***Working memory*** was assessed using spatial working memory (SWM) test, which is used to measure the ability to retain spatial information and manipulate items stored in working memory, problem solving and self-organized search strategies. During the SWM test, the participants were presented with 3, 4, 6, 8, or 12 randomly distributed colored boxes on the screen. After that, the participants were supposed to search for tokens hidden in the boxes. When a token was found, it was supposed to be moved to fill an empty panel on the right-hand side of the screen. Once the token had been removed from the box, the participant had to recall that the computer would never hide a new token in a box that previously contained one; therefore, the participants were not supposed to revisit the same boxes.

***Information processing*** was evaluated using rapid visual information (RVP) test, which is used to assess visual processing, recognition, and sustained attention. In this test, the participant was presented with three number sequences (3–5–7, 2–4–6, and 4–6–8) next to a large box where the number 1–9 appeared in a random order at a rate of 100 numbers per minute. Whenever any of the particular sequences were presented, the participant was supposed to press a touchscreen button. Altogether, nine target sequences were presented at 100-s intervals during the 6-min assessment phase. During the practice phase, the participant was given visual cues (i.e., colored or underlined numbers) to help him or her recognize the particular sequence. At the assessment phase, the cues were no longer presented.

***Reaction time*** was evaluated using reaction time (RTI) test, which assesses the speed of response and movement as well as accuracy on a task where the stimulus was unpredictable (five-choice location task). Five large circles were presented on the screen, and the participant was supposed to press down a touchscreen button at the bottom of the screen and wait until a small yellow spot appeared in any of the five large circles. When the yellow spot appeared, the participant was supposed to touch the yellow spot as soon as possible with the same hand that was pressing the touchscreen button.

### Age and education

Age was defined in full years at the end of the year 2018. Education years were queried from all participants aged 18 years and older. In Finland, the mean age at which secondary education is completed is 28 years. According to our data, 492 (92%) of the 533 participants who reported studying were at most 28 years old. Thus, we considered the own education of the participants aged 28 years and older. For participants aged 18–28 years, their own education was provided if the participant did not report full- or part-time studying. For all participants aged under 18 years and participants aged 18–28 years who reported studying, parental education years were considered (the maximum years of parental education for those participants with data for both parents). Hence, for adults (excluding students), education reflects self-acquired cognitive reserve, while for under 18-year-old participants and students, parental education reflects socioeconomic status.

### Statistical methods

The CANTAB^®^ test produced several variables, the detailed information of which is presented in Online Resource 1. We used Flury’s common principal component analysis [22] to derive the principal component scores for (i) across all domains/whole CANTAB® test battery and (ii) separately for each measured cognitive domain/each of the four separate subtests. The main idea of this method is to conduct a principal component analysis for a dataset arranged in multiple groups. This approach allows the groups to have different means, variances, and correlations but assumes that the principal components are the same in those groups. Given that cognitive performance may differ between generations and among participants of different ages, we defined six groups as the input for the analysis: G0, G1, G2 (aged 25–37 years; adulthood), G2 (18–24 years; young adulthood), G2 (13–17 years; adolescence) and G2 (7–12 years; childhood). The G2 generation (children of the original YFS participants) was divided into four age windows because we wanted to recognize the possible variation in cognitive performance within different developmental phases. We standardized the variables of cognitive performance before the analysis and subsequently windsorized a few outlying values (> 10 SDs from the mean) to ± 10 to control for any disproportionate influence. Common principal component analysis was implemented with the multigroup package in R (version 4.1.3). Participants’ first, second, and third principal component scores were extracted, and the first principal component scores were chosen to represent overall cognition as well as cognitive domains representing learning and memory, working memory, information processing, and reaction time. This selection was based on the largest amount of explained variance compared to the following principal components as well as reasonable variable loadings of the relevant key variables within each cognitive test. The sign of each principal component was assigned so that higher principal component scores indicate better test performance (e.g., less errors, shorter latency times, and better accuracy). For reaction time, the distribution of participants’ first principal scores was skewed, while for the other domains, the first principal scores were normally distributed according to visual inspection.

We estimated the means of cognitive performance in different domains across the age range of 7–92 years nonparametrically by Loess smoothing, which uses locally weighted polynomial regression in the estimation of the smooth fit. Visualization of the cognitive performance trajectories was based on these estimates. For the subsequent analyses, the first principal component scores for each domain were standardized such that 20–29-year-old participants (20 ≤ age < 30) had a mean value of 0 and standard deviation of 1, as cognitive performance was observed to be at its highest during this age period based on the visualization. Therefore, the values reported for each generation describe how many standard deviations the given principal component scores differ from those of 20–29-year-old participants. In addition, cognitive performance was visualized in similar manner using the second and third principal component scores, and additional analyses were conducted if visual inspection suggested age-related changes in these components.

The associations between age, sex, and education and overall cognitive performance and the specific cognitive domains were studied using generation-specific linear models. Furthermore, for the G2 generation, models were created for each of the four age groups to capture the increasing developmental trajectory of cognitive performance at a young age. The exposure variables age, sex, and education were entered simultaneously into the cognitive domain-specific models. Age was first linearly represented, after which a quadratic term for age was applied (age2) to allow for a nonlinear association between age and cognitive performance (as suggested by Loess fits). Model residuals were homoscedastic and normally distributed by visual inspection except for reaction time, the distribution was skewed. Therefore, we tested the 1/x transformation for reaction time. Due to easier interpretability, untransformed results are shown in Tables 3 and 4. Linear models were generated using SAS 9.4 (SAS Institute, Cary, North Carolina, USA) software, and the level of statistical significance was set at *p* ≤ 0.05.

We estimated intergenerational correlations among the first principal component scores representing each cognitive domain by calculating partial Pearson’s correlation (partial Spearman’s rank correlation for reaction time) between G0 and G1, G1 and G2, and G0 and G2. The analyses were stratified for sex and adjusted for age. Hence, we obtained age-adjusted correlations between mothers and daughters, mothers and sons, fathers and daughters, and fathers and sons in G0 and G1 as well as in the G1 and G2 generations. For G0 and G2, sex-specific and age-adjusted correlations were calculated between grandparents and grandchildren. The intergenerational correlation analyses were conducted using the ppcor package (version 1.1) in R. Correlations with *p* ≤ 0.05 were considered to be statistically significant.

## Results

### Characteristics of the study population

**Fig. 1:**
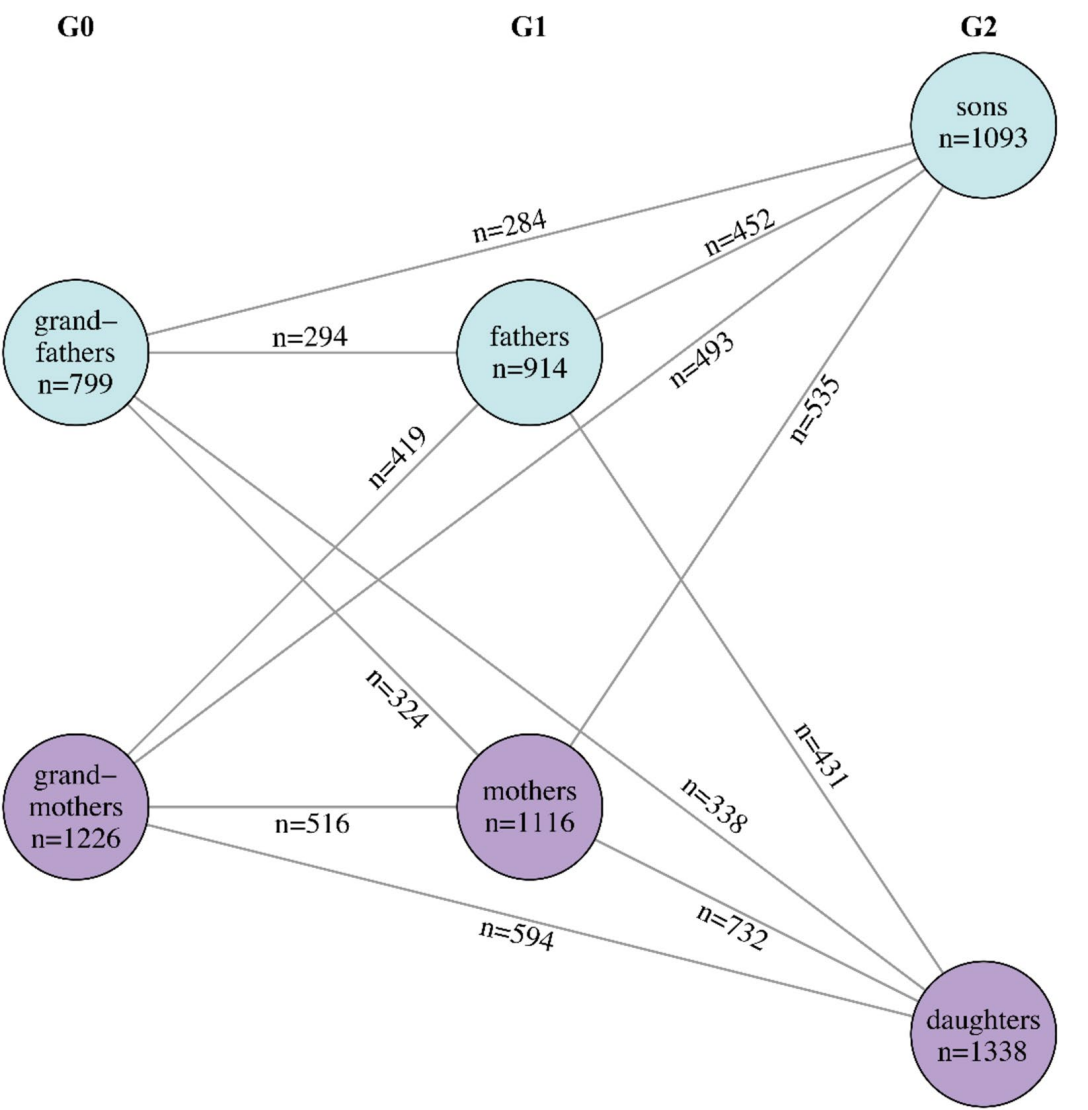
Number of participants within each generation G0, G1, and G2. The blue circles represent males, and the purple circles represent females. The numbers along the edges denote the numbers of the respective dyads

In total, 6486 individuals completed cognitive testing successfully in at least one cognitive domain. The participants’ cognitive data were missing for (i) technical reasons (*n* = 33), (ii) unwillingness to participate in some of the tests (*n* = 776), (iii) distraction caused by a study nurse or the environment (*n* = 11), and (iv) unknown reasons (*n* = 96). The number of participants for each generation and sex, as well as the number of parent/offspring and grandparent/grandchild dyads, are shown in Fig. 1. The characteristics of the three-generational study population, generation-specific characteristics, and domain-specific numbers of participants with cognitive performance data are presented in Table 1. Within the complete three-generational population, the mean age was 45.3 years (range 7.0–92.0), and 56.7% were females. The mean age of generation G1, the original YFS participants, was 48.7 years (range 41.0–56.0), while in generation G0 consisting of their parents, the mean age was 72.7 years (range 59.0–92.0), and in generation G2 consisting of their children, the mean age was 19.7 years (range 7.0–37.0). Within each generation, females participated more actively than males; 55.0% of the original YFS participants, 60.5% of their parents, and 55.0% of their offspring were females. Corresponding data within the G2 generation age groups are presented in Table 2.

**Table 1:** Study population characteristics by generation

	All participants (*n* = 6486)		G0 (*n* = 2025)		G1 (*n* = 2030)		G2 (*n* = 2431)	
**Age, years**	45.3 (7.0–92.0)		72.7 (59.0–92.0)		48.7 (41.0–56.0)		19.7 (7.0–37.0)	
**Sex, male**	2803 (43.3)		799 (39.5)		914 (45.0)		1090 (45.0)	
**Education, years**	14.3 (4.0)		11.9 (4.1)		15.8 (3.7)		15.2 (3.3)	
**Data on cognitive performance**	***N*** **(%)**	**Mean (SD)**	***N*** **(%)**	**Mean (SD)**	***N*** **(%)**	**Mean (SD)**	***N*** **(%)**	**Mean (SD)**
Overall cognitive performance	5570	− 1.12 (1.51)	1422	− 2.87 (0.97)	1867	− 0.92 (1.17)	2281	− 0.19 (1.02)
Learning and memory (*PAL test*)	6215	− 1.05 (1.48)	1870	− 2.51 (1.03)	1951	− 0.92 (1.16)	2394	− 0.00 (1.01)
Working memory (*SWM test*)	6386	− 0.86 (1.19)	1937	− 1.84 (0.75)	2022	− 0.61 (1.13)	2427	− 0.27 (1.02)
Information processing (*RVP test*)	5773	− 0.46 (1.27)	1534	− 1.33 (1.24)	1932	0.15 (1.03)	2307	− 0.40 (1.14)
Reaction time (*RTI test*)*	6366	0.55 (1.15)	1937	0.75 (0.61)	2016	0.62 (0.37)	2413	0.40 (1.54)

The numbers are presented as the means (ranges) for continuous variables and as numbers of participants and percentages for categorical variables. Self-reported education years were considered for the G1 and G0 participants. For the G2 participants, self-reported education years were applied for all participants aged ≥ 28 years. For the participants aged ≤ 18 years, a maximum of parental education years was applied. For the G2 participants aged 19–28 years, parental education years were applied if the participant reported that he/she was studying; otherwise, self-reported education years were used. The cognitive performance variables are standardized domain specifically in relation to participants aged 20–29 years. Hence, the means (SD) describe how many standard deviations the given principal component differs from the highest values reached at age 20–29 years

^*^Due to its nonnormal distribution, the median (interquartile range) is reported for the reaction time (RTI test)

**Table 2:** Characteristics of the study population within the G2 generation by age group

	G2 (age 25–37 years) (*n* = 662)		G2 (age 18–24 years) (*n* = 821)		G2 (age 13–17 years) (*n* = 487)		G2 (age 7–12 years) (*n* = 461)	
**Age, years**	28.3 (2.8)		21.2 (2.0)		14.8 (1.4)		9.9 (1.7)	
**Sex, male**	293 (44.3)		327 (39.8)		253 (52.0)		217 (47.7)	
**Education, years**	15.0 (2.6)		13.9 (2.8)		16.1 (3.6)		16.5 (3.7)	
**Data on cognitive performance**	**N (%)**	**Mean (SD)**	**N (%)**	**Mean (SD)**	**N (%)**	**Mean (SD)**	**N (%)**	**Mean (SD)**
Overall cognitive performance	646	− 0.11 (1.03)	802	0.01 (1.01)	471	− 0.15 (0.91)	362	− 0.83 (0.93)
Learning and memory (*PAL test*)	656	− 0.15 (1.04)	810	0.04 (1.02)	480	0.24 (0.87)	448	− 0.14 (1.02)
Working memory (*SWM test*)	662	− 0.05 (1.02)	821	− 0.01 (1.00)	487	− 0.37 (0.87)	457	− 0.98 (0.82)
Information processing (*RVP test*)	650	0.01 (1.02)	814	− 0.05 (1.00)	477	− 0.69 (0.96)	366	− 1.53 (1.00)
Reaction time (*RTI test*)*	658	0.13 (0.92)	820	− 0.05 (1.02)	486	− 0.43 (1.34)	449	− 0.83 (2.70)

The numbers are presented as the means (ranges) for continuous variables and as numbers of participants and percentages for categorical variables. Self-reported education years were applied for all participants aged ≥ 28 years. For the participants aged ≤ 18 years, a maximum of parental education years was applied. For the G2 participants aged 19–28 years, parental education years were applied if the participant reported that he/she was studying; otherwise, self-reported education years were used. The cognitive performance variables are standardized domain specifically in relation to participants aged 20–29 years. Hence, the means (SD) describe how many standard deviations the given principal component differs from the highest values reached at age 20–29 years

^*^Due to its nonnormal distribution, the median (interquartile range) is reported for the reaction time (RTI test)

### Common principal component analyses

**Fig. 2:**
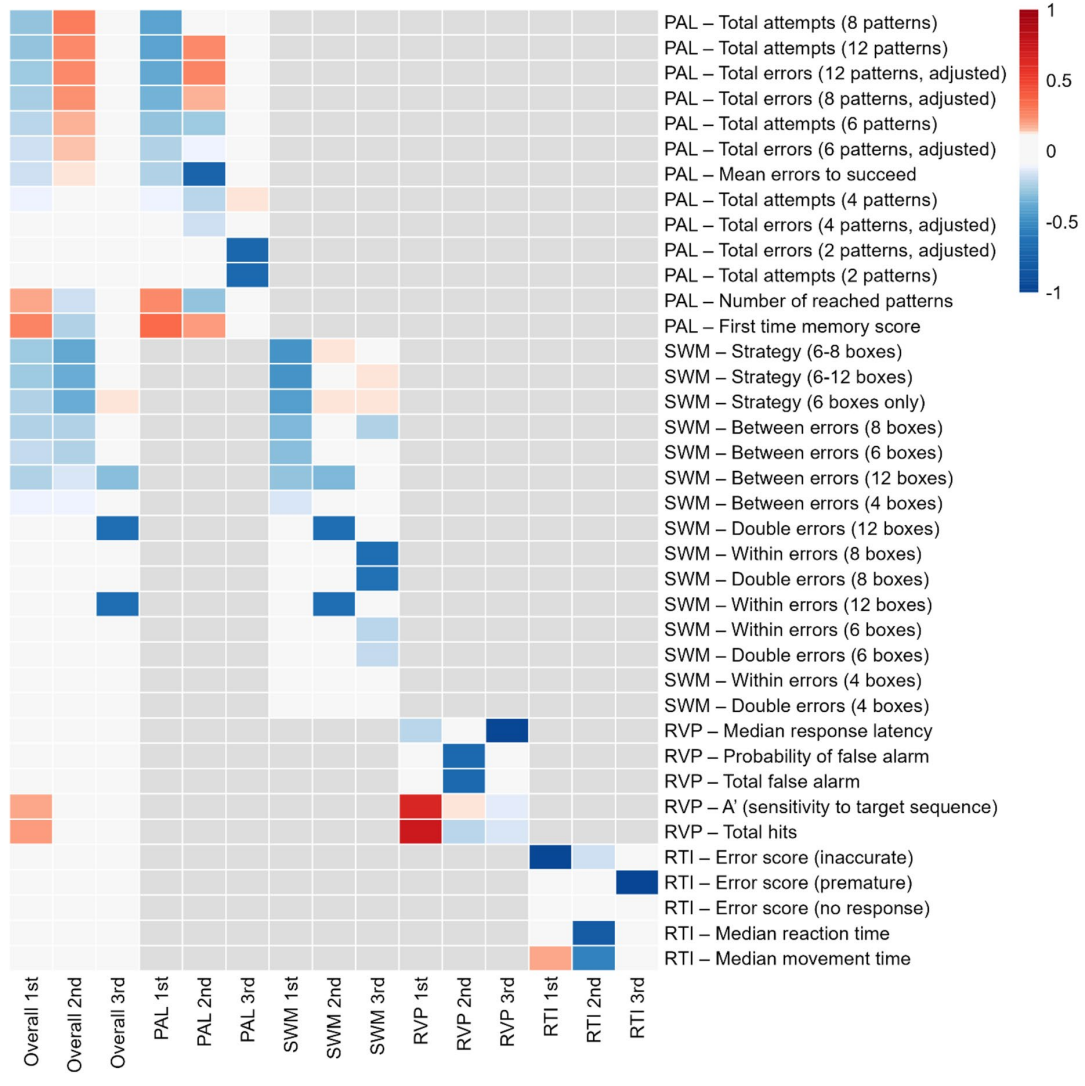
Loadings within the first three principal components for overall cognition and for domain-specific principal components. The rows represent individual test variables obtained from CANTAB®, and the columns represent the principal components. Blue cells indicate negative and red cells indicate positive loadings according to the legend. *PAL* learning and memory, *SWM* working memory, *RVP* information processing, *RTI* reaction time

The first three principal components from the analyses for the whole cognitive test battery represented 36% of the variation of the entire test. Approximately, half of the variation was attributed to the first principal component (18%, Online Resource 1). The first principal component representing overall cognition within our test battery was a combination of the components of learning and memory (PAL), working memory (SWM) and information processing (RVP) domains: first-time memory score (PAL test) and total hits (RVP test) had the highest positive coefficient for the first principal component, whereas a number of errors and attempts (8 and 12 patterns; PAL test) and strategy (SWM test) tended to have the strongest negative coefficients (Fig. 2). The domain-specific loadings within the first three principal components and the rate of variation they represented are shown in Online Resource 2. For learning and memory, the first principal component represented 46% of the variation. The number of reached patterns and first-time memory score had the highest positive coefficients, while the strongest negative coefficients were observed for total attempts (8 and 12 patterns). For working memory, the first principal component represented 33% of the variation. No variable had positive coefficients, and the strongest negative coefficients were those indicating strategy use, followed by between-error scores. For information processing, the first principal component represented 47% of the variation. Total hits and A’ (an indicator of sensitivity to the target sequence) had the highest positive coefficients, while median response latency was the variable with the strongest negative coefficient. Finally, for the reaction time, the first principal component represented 33% of the variation. The median movement time had the highest positive coefficient, while the error score due to inaccuracy had the strongest negative coefficient.

### Visualization of cognitive performance trajectories

**Fig. 3:**
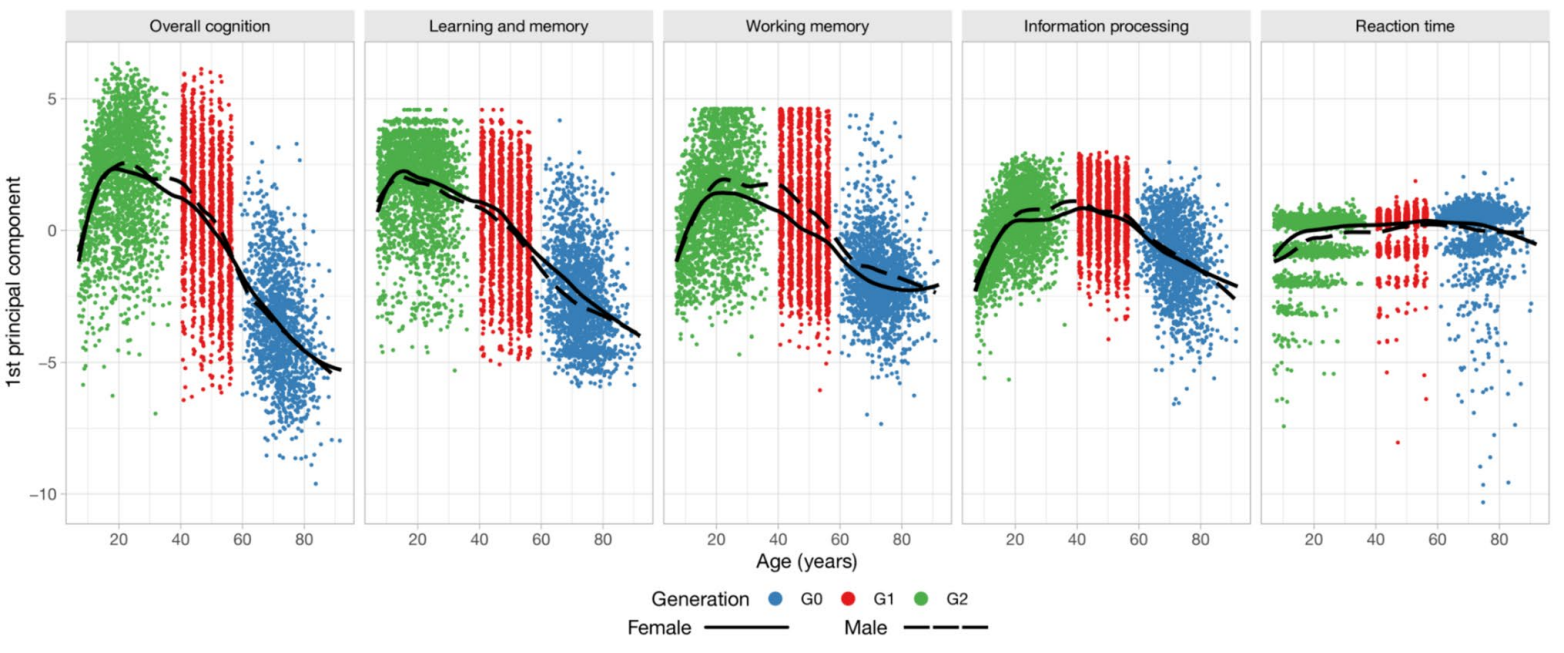
Cognitive performance trajectories between ages 7 and 92 years. The data points represent the first principal component scores for each cognitive domain, colored by the generation according to the legend. The trajectories for females and males were obtained by Loess smoothing

The cognitive performance trajectories of males and females aged 7–92 years are visualized in Fig. 3. In general, the trajectories indicated the rapid development of cognitive performance during childhood and adolescence and a decline in cognitive performance toward older age. However, the trajectories differed between cognitive domains. Learning and memory reached its peak before the age of 20 years and then started to decline with an increasing slope in old age. Working memory and information processing increased steeply at a young age but remained stable or even increased during adulthood and decreased gradually during old age. Reaction time had a distinctive trajectory, remaining rather stable after early age, with a surprisingly mild decline in old age. As overall cognition was explained mostly by the components of learning and memory, working memory, and information processing, its trajectory resembled these curves. To confirm the nonlinear association of age observed via visualization, the quadratic term of age was statistically significant (*p* < 0.001) for all cognitive domains (models not shown). By visual inspection, sex affects cognitive performance, but the differences appear to be modest.

The cognitive performance trajectories based on the participants’ second and third principal component scores are visualized in Online Resource 3. For reaction time, the second principal scores, affected mostly by the negative loadings of median reaction time and median movement time (Fig. 2), increased rapidly during the childhood and declined gradually towards old age (Online Resource 3). For the other cognitive domains, the second and third principal component scores remained rather steady, indicating that the essential information related to age is extracted in the first principal component scores.

### Role of age, sex, and education on cognitive performance

To formally test the role of age, sex, and education on cognitive performance, we utilized linear models within each generation to further explore the role of these determinants on cognitive performance. In concordance with the visualization, age was negatively associated with most of the cognitive domains in adult generations G0 and G1 (Table 3). In generation G2, which included children, adolescents, and young adults, the association between age and cognitive performance was positive except for that between learning and memory (Table 3). Since generation G2 included a wide age range of participants, they were further analyzed in four age groups to investigate the development of cognitive performance at a young age (Table 4). In general, the results highlighted the early development of cognitive performance in childhood (7–12 years) and adolescence (13–17 years), reaching a plateau in young adulthood (18–24 years) and starting to decrease thereafter (25–37 years). Learning and memory developed early and reached a plateau during adolescence, while information processing still matured during adolescence and reached a plateau in young adulthood and adulthood (Table 4).

**Table 3:** Linear associations between age, sex and education and cognitive domains within the generations

		G0 (59–92 years)	G1 (41–56 years)	G2 (7–37 years)
Cognitive domain		β estimate (95% CI)	β estimate (95% CI)	β estimate (95% CI)
**Overall cognition**	Age, years	− **0.05 (**− **0.06, **− **0.04)**	− **0.07 (**− **0.08, **− **0.06)**	**0.03 (0.03, 0.04)**
	Female sex	0.05 (− 0.05, 0.15)	− **0.25 (**− **0.35, **− **0.15)**	− 0.03 (− 0.12, 0.06)
	Education, years	**0.04 (0.03, 0.06)**	**0.06 (0.05, 0.08)**	**0.03 (0.01, 0.04)**
**Learning and memory**	Age, years	− **0.05 (**− **0.05, **− **0.04)**	− **0.06 (**− **0.07, **− **0.05)**	− 0.00 (− 0.01, 0.00)
	Female sex	**0.28 (0.19, 0.38)**	0.09 (− 0.01, 0.20)	**0.12 (0.03, 0.20)**
	Education, years	**0.04 (0.03, 0.05)**	**0.06 (0.04, 0.07)**	**0.03 (0.02, 0.04)**
**Working memory**	Age, years	− **0.02 (**− **0.03, **− **0.01)**	− **0.05 (**− **0.06, **− **0.04)**	**0.05 (0.04, 0.05)**
	Female sex	− **0.23 (**− **0.30, **− **0.16)**	− **0.49 (**− **0.59, **− **0.39)**	− **0.11 (**− **0.19, **− **0.03)**
	Education, years	**0.01 (0.00, 0.02)**	**0.03 (0.02, 0.05)**	0.01 (− 0.00, 0.02)
**Information processing**	Age, years	− **0.06 (**− **0.07, **− **0.04)**	− **0.02 (**− **0.03, **− **0.01)**	**0.08 (0.07, 0.09)**
	Female sex	− 0.07 (− 0.19, 0.05)	− **0.23 (**− **0.32, **− **0.14)**	− **0.20 (**− **0.29, **− **0.11)**
	Education, years	**0.07 (0.05, 0.08)**	**0.08 (0.06, 0.09)**	**0.03 (0.02, 0.04)**
**Reaction time**	Age, years	− **0.02 (**− **0.03, **− **0.01)**	**0.02 (0.01, 0.03)**	**0.05 (0.04, 0.06)**
	Female sex	**0.21 (0.07, 0.35)**	**0.25 (0.16, 0.33)**	**0.52 (0.41, 0.62)**
	Education, years	0.00 (− 0.02, 0.02)	− 0.00 (− 0.01, 0.01)	− **0.03 (**− **0.05, **− **0.02)**

The principal components of cognitive performance were standardized in relation to the age group 20–29 years (20 ≤ age < 30). Hence, the β estimates describe how many standard deviations (95% confidence intervals) the given principal component differs from the highest values reached at age 20–29 years

**Table 4:** Linear associations between age, sex and education with cognitive domains within the G2 generation in different age groups reflecting adulthood, young adulthood, adolescence, and childhood

		G2 (25–37 years)	G2 (18–24 years)	G2 (13–17 years)	G2 (7–12 years)
Cognitive domain		β estimate (95% CI)	β estimate (95% CI)	β estimate (95% CI)	β estimate (95% CI)
**Overall cognition**	Age, years	− **0.05 (**− **0.08, **− **0.02)**	− 0.03 (− 0.07, 0.01)	**0.11 (0.05, 0.17)**	**0.25 (0.19, 0.31)**
	Female sex	− 0.09 (− 0.25, 0.08)	− 0.12 (− 0.27, 0.03)	0.05 (− 0.12, 0.23)	0.02 (− 0.16, 0.21)
	Education, years	**0.06 (0.03, 0.09)**	**0.07 (0.05, 0.10)**	**0.028 (0.00, 0.05)**	0.02 (− 0.00, 0.05)
**Learning and memory**	Age, years	− **0.04 (**− **0.07, **− **0.02)**	− 0.01 (− 0.05, 0.03)	0.02 (− 0.04, 0.08)	**0.13 (0.08, 0.19)**
	Female sex	**0.18 (0.01, 0.34)**	0.14 (− 0.01, 0.29)	0.09 (− 0.07, 0.26)	0.04 (− 0.15, 0.23)
	Education, years	**0.05 (0.02, 0.08)**	**0.05 (0.02, 0.08)**	0.01 (− 0.01, 0.04)	0.02 (− 0.00, 0.05)
**Working memory**	Age, years	− **0.03 (**− **0.06, **− **0.00)**	− **0.04 (**− **0.08, **− **0.00)**	**0.11 (0.05, 0.16)**	**0.17 (0.13, 0.21)**
	Female sex	− **0.23 (**− **0.40, **− **0.07)**	− **0.26 (**− **0.41, **− **0.11)**	0.02 (− 0.14, 0.18)	0.06 (− 0.09, 0.21)
	Education, years	0.025 (− 0.01, 0.06)	**0.06 (0.03, 0.08)**	**0.02 (0.00, 0.04)**	0.01 (− 0.01, 0.03)
**Information processing**	Age, years	0.00 (− 0.02, 0.03)	0.01 (− 0.03, 0.04)	**0.21 (0.15, 0.27)**	**0.27 (0.20, 0.33)**
	Female sex	− **0.41 (**− **0.57, **− **0.25)**	− **0.30 (**− **0.45, **− **0.16)**	− 0.02 (− 0.19, 0.16)	− 0.14 (− 0.33, 0.05)
	Education, years	**0.10 (0.07, 0.13)**	**0.08 (0.06, 0.11)**	**0.03 (0.01, 0.05)**	**0.03 (0.00, 0.06)**
**Reaction time**	Age, years	0.02 (− 0.00, 0.05)	0.01 (− 0.03, 0.05)	**0.09 (0.01, 0.18)**	**0.12 (0.01, 0.22)**
	Female sex	**0.33 (0.19, 0.48)**	**0.41 (0.26, 0.56)**	**0.74 (0.50, 0.98)**	**0.68 (0.33, 1.04)**
	Education, years	0.00 (− 0.02, 0.03)	− 0.01 (− 0.04, 0.02)	− **0.05 (**− **0.08, **− **0.02)**	− 0.05 (− 0.10, 0.00)

The principal components of cognitive performance were standardized in relation to the age group 20–29 years (20 ≤ age < 30). Hence, the β estimates describe how many standard deviations (95% confidence intervals) the given principal component differs from the highest values reached at age 20–29 years

Female sex was associated with higher values for learning and memory and reaction time but lower values for working memory in all three generations (Table 3, visualized in Fig. 3). Male sex was associated with greater information processing in G1 and G2 but not in the oldest G0 generation. Overall cognition was lower in females only in the adult G1 generation, but there was no sex difference in cognition between the younger G2 generation and the older G0 generation (Table 3). When investigating sex differences in different age groups within generation G2, better performance of males in working memory and information processing and better performance of females in learning and memory started to become visible during young adulthood (Table 4). Reaction time was greater in females already in childhood (Table 4).

In generations G0 and G1, the association between education and cognitive performance was positive and similar but opposite in magnitude to the estimates of age in all the other cognitive domains except for reaction time, which was not associated with education (Table 3). In the whole G2 generation, education (parental education for participants younger than 18 years and students) was positively associated with overall cognition, learning and memory, and information processing (Table 3). When assessing the effect of education within G2 age groups, parental education was positively associated with information processing but negatively associated with reaction time in childhood (Table 4). However, during adolescence, parental education was positively associated with overall cognition and working memory in addition to information processing, and a negative association with reaction time persisted during adolescence. In young adulthood and adulthood, education was assessed as the participant’s own education (parental education for 18–28-year-old participants who were students), and the role of education in cognitive performance resembled that of adult generations G0 and G1: education was positively associated with cognitive performance in all domains except for reaction time, which was not associated with education (Table 4).

Finally, additional analyses were performed for reaction time. First, as the distribution of reaction time was skewed, the above analysis was repeated using 1/x transformation. With 1/x transformation, the association of sex was statistically significant only in the G2 generation, especially during adolescence and young adulthood (Online Resource 4). Second, as the visualization of the second principal component scores revealed age-related changes in reaction time (Online Resource 3), similar linear models as above were created using second principal component scores of reaction time which reflects reaction and movement time. In this analysis, reaction time increased rapidly in childhood, and started to decline in adulthood towards old age mostly without differences between sexes (Online Resource 5).

### Intergenerational correlations of cognitive performance

**Fig. 4:**
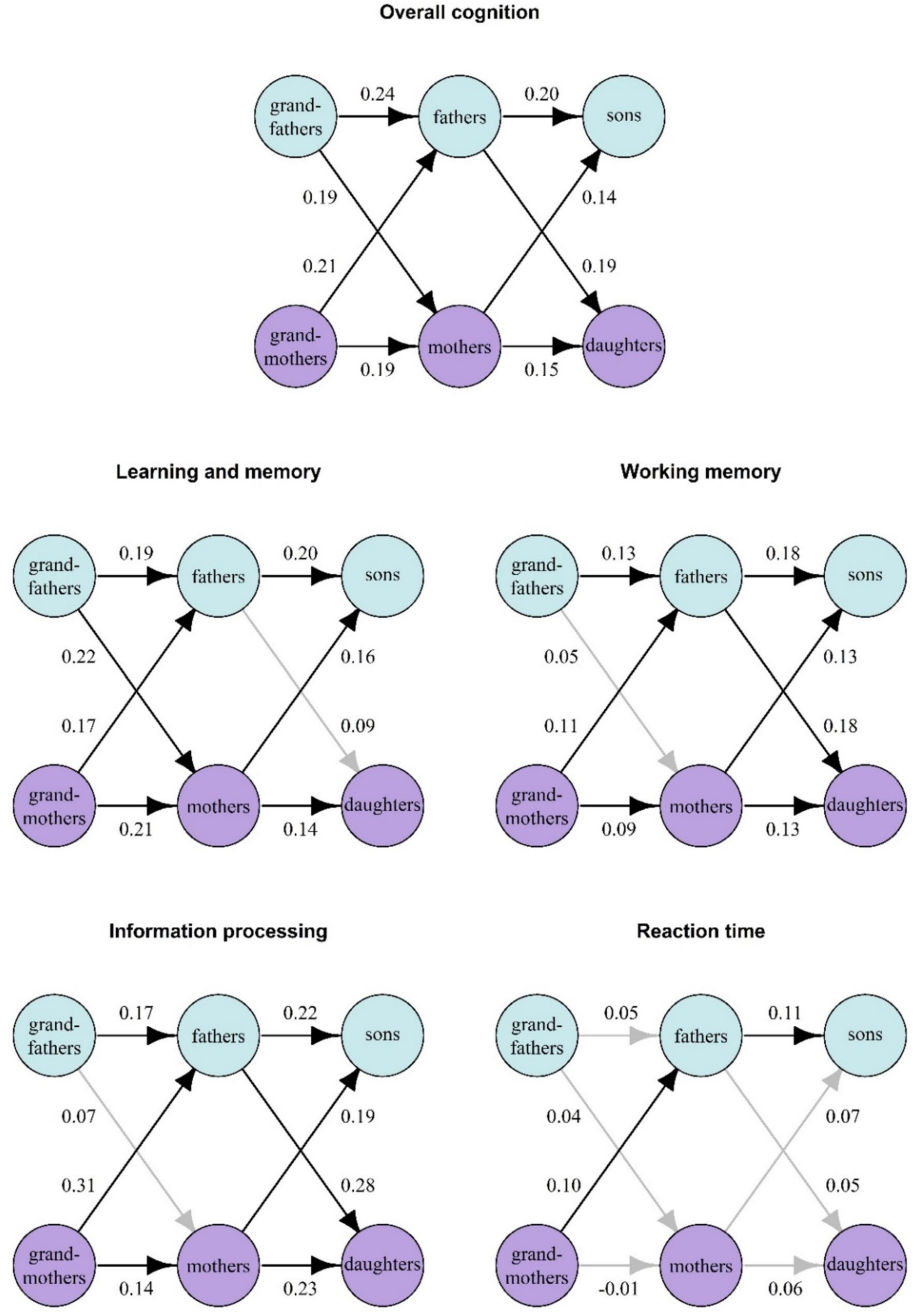
Intergenerational correlations between family members within each cognitive domain. The blue circles represent males, and the purple circles represent females. The numbers next to the arrows are age-adjusted Pearson’s correlations (Spearman correlations for reaction time). Statistically significant correlations are shown as black arrows, and nonsignificant correlations are shown as gray arrows

The age-adjusted intergenerational correlations of cognitive performance stratified by sex are presented in Fig. 4. For overall cognition, the correlation coefficients varied between 0.14 and 0.24, and all the correlations were statistically significant. Correlations mostly of similar magnitude were observed for learning and memory, information processing, and working memory. The highest correlation, *r* = 0.31 (*p* < 0.001), was observed in information processing between G0 mothers and their G1 sons. For reaction time, correlations between family members were mostly close to zero. The correlations between G0 and G1 and between G1 and G2 were relatively similar in magnitude, suggesting that age has no major effect on intergenerational correlations between family members. Correlation coefficients between G0 and G1 are reported in detail in Online Resource 6, and those between G1 and G2 are reported in Online Resource 7.

Corresponding age-adjusted sex-specific correlation coefficients between grandparent G0 and grandchild G2 are reported in Online Resource 8. Between these two generations, the correlations were weak. Statistically significant correlations were observed only in learning and memory between grandmothers and grandsons (*r* = 0.11, *p* = 0.020) and grandfathers and grandsons (*r* = 0.17, *p* = 0.009) and in information processing between grandmothers and grandsons (*r* = 0.11, *p* = 0.049).

For reaction time, correlations were additionally calculated using the second principal component scores reflecting reaction and movement time. Weak but statistically significant correlations were observed between G1 and G2, but not between the other generations (Online Resource 9).

## Discussion

This study aimed to explore (i) the effect of age at different stages of life and (ii) intergenerational associations across cognitive domains. We utilized a computerized neurocognitive testing platform, CANTAB®, to assess cognitive performance with an identical testing procedure for all age groups in our epidemiologic cohort study, which included participants in three familiarly related generations. We used common principal component analysis to summarize the rich raw data obtained from the CANTAB® test battery to form a single measure of overall cognition as well as of cognitive domains related to learning and memory, working memory, information processing, and reaction time. Trajectories of cognitive performance across lifespan differed between cognitive domains. While intergenerational correlations existed between two consecutive generations, they were mostly weak and lacked systematic sex-specific transmission.

We have previously shown that principal component analysis is a valid data reduction method for analyzing CANTAB^®^ data [23]. In the present work, we extended this idea and used common principal component analysis with specific age groups to allow different means and standard deviations for each age group, reflecting differences in gains and losses of cognitive performance in different stages of life. The benefit of this method is that by extracting the first principal component, we obtained a single test score describing cognitive performance in each cognitive domain rather than reporting multiple different error scores and latency times. In the present dataset, we observed known sex-specific differences, such as females’ better performance in learning and memory and males’ better performance in information processing and working memory [24–26]. Furthermore, we observed that education is positively associated with cognitive performance, which is in line with the findings of previous studies indicating that years of education enhance cognitive reserve [27, 28]. Parental education was positively associated especially with information processing and working memory, supporting the literature on the positive effect of parental education and socioeconomic status on offspring’s cognitive performance [29]. As sex and education are two well-known determinants of cognitive performance, replicating these known results with our dataset further validates the applicability of common principal component analysis to CANTAB^®^ data. While the first principal component of the whole dataset describing overall cognition within our test battery explained only 18% of the variation, domain-specific explained variations were greater: 33% for both reaction time and working memory, 46% for learning and memory, and 47% for information processing. Therefore, we focus on domain-specific results.

It is well known that cognitive performance changes during the lifespan, but the information is mostly gathered from separate study populations in different age groups with differing methods of neurocognitive assessment. Cognitive performance is studied mostly from the perspective of general cognitive ability, which has been schematically described as an inverted U-curve [7]. In recent years, specific cognitive domains have been studied in more detail, and it has become evident that different cognitive domains both develop and decline at varying rates, modifying the shape of the developmental curve differently across cognitive domains [2, 3]. In the present study, we observed a rise in cognitive performance at an early age, reaching a plateau around young adulthood, and a decline toward old age, suggesting that our data roughly follow the inverted U-curve. However, the shapes of the cognitive trajectories differed between cognitive domains. Learning and memory developed rapidly in childhood, peaked in adolescence, and started to decrease already in young adulthood, with an increasing slope toward old age. Working memory and information processing matured later and were at their highest levels during adulthood with longer plateaus, decreasing more gradually toward older age. These results are in line with observations derived from traditional psychometric tests [30]. Fluid cognitive abilities, which refer to the ability to learn and solve new problems without previously acquired knowledge, mature early and start to decline around mid-twenties onward, whereas crystallized cognitive abilities, which depend on learning and cultural influences and reflect experience based on acquired knowledge, such as information processing, mature later and continue to improve in adulthood [31]. Domain-specific trajectories in cognitive performance resemble heterochronic development and deterioration of gray matter volume, for which the peak of the inverted U-curve occurs at different age for different regions of cerebral cortex [7, 32, 33]. Similar cognitive domain-specific findings were also reported among the Dutch population covering almost the whole lifespan [10]. The findings of the present cross-sectional study across the lifespan are also in line with our recent longitudinal study among the original YFS participants (G1 cohort in the present study), in which we reported that age is associated with the change in cognitive performance so that those in late midlife have experienced a greater decline compared to those in their forties, supporting the nonlinear effect of age observed in the present study [34].

Surprisingly, the trajectories of reaction time remained relatively stable during the lifespan when considering the first principal component scores. This could be partly explained by the test variables loaded in the first principal component, which were error scores due to inaccuracy and median movement time. Thus, the first component reflects not only reaction time, as error-free performance is rewarded in its computation. In the second principal component, the median reaction and movement time were loaded more compared to measures of accuracy, and an increase at young age and a decrease towards old age was observed. Our result obtained using the second principal component is in line with the study among the Dutch population, in which the rate of age-related decline was particularly strong for measures of cognitive speed [10]. The simple reaction time increases with age but is only approximately two milliseconds in a decade [35]. On the other hand, older adults may favor accuracy over speed, whereas younger individuals aim to balance speed and accuracy [36]. The first principal components of reaction time in the present study included both speed and accuracy, which may explain the relatively stable trajectory across the lifespan observed in our study, possibly representing different strategies at different ages for tasks involving speed-accuracy compromise.

The effect of sex was mostly in agreement with the literature [24–26] as well as with our previous study among the original YFS population (G1): females outperformed males in memory and learning, while males performed better in working memory and information processing [23]. However, according to visual inspection of the sex-specific trajectories, the differences between sexes were mostly modest, and they started to become visible in adolescence or young adulthood. This finding is in line with previous literature reporting that while there may be sex-specific differences during cognitive development, these differences are usually small [1, 37].

The heritability of cognitive performance has been studied mostly in twin and adoption studies. While traditional research has focused on general cognitive function, more recent studies have examined different cognitive domains. A recent large meta-analysis reviewing monozygotic–dizygotic comparisons concluded that the average heritability across different specific cognitive domains was 56%, similar to that of general cognitive function [16]. However, the magnitude of heritability varies widely across cognitive domains [10, 16]. In addition, while the heritability of general cognitive function increases from approximately 20% in infants to 60% in adults, specific cognitive domains do not show a similar developmental increase, and in some domains, heritability decreases with increasing age [16]. In the present study, the intergenerational correlations between two consecutive generations were mostly statistically significant but weak. In addition, the correlations between grandparents (G0) and parents (G1) as well as between parents (G1) and their offspring (G2) were similar in magnitude, suggesting that intergenerational correlations remain roughly the same at different ages. To our knowledge, there are no prior large-scale population studies investigating cognitive performance in three familiarly related generations. In the Dutch study, most of the participants were adolescent twin pairs, but other family members were also included [10]. When all available pedigree information was used in their study, genetic factors accounted for between 13 and 49% of the total variance in cognitive speed and accuracy [10]. However, the higher heritability of these findings compared to our findings may be attributed to the considerable portion of twins in their dataset. In a smaller study comprising families at risk of developing substance use disorder, correlations of executive function between adolescents and their parents ranged between 0.11 and 0.43 [38]. The executive function of parents was also associated with that of their offspring in a Chinese cohort [18] and in a US cohort even after adjusting for socioeconomic status [19]. A larger German study further suggested that intergenerational cognitive performance transmission is sex specific [17]. Accordingly, a Swedish study investigating the odds of students receiving top marks in school subjects reported higher odds, especially if their grandfathers did well in school, whereas their grandmothers had a smaller role in intergenerational transmission [20]. While some of the correlations varied in magnitude between sexes in our present study, we did not observe systematic sex-specific transmission in any cognitive domain. Moreover, when assessing associations of cognitive performance between grandparents and their grandchildren, correlations were mostly nonsignificant. Taken together, our data summarizing cognitive performance within each domain by principal component analysis suggest that while intergenerational correlations are low, they do exist. However, cognitive abilities are likely affected by many factors in addition to heritable components.

The strength of the present study is the unique three-generation study population with more than 2000 individuals in each of the three familiarly related generations, representing both sexes and covering a wide age range. All the participants were assessed using the same computerized neurocognitive platform, CANTAB®, at the same time point, which enabled us to study the effects of age with comparable methodology across the study population. While the CANTAB® is not yet widely used in clinical practice, it could be utilized as a fast and cost-effective platform to screen for possible cognitive impairments due to aging-related memory diseases, such as Alzheimer’s disease but also in younger populations due to, e.g., mood and anxiety disorders [39] and attention-deficit/hyperactivity disorders [40]. Studies on the general population are needed to describe the natural development and deterioration of cognitive performance, which may facilitate the identification of the critical time windows for the promotion of cognitive health and early prevention of cognitive decline. While the present study describes trajectories and variation in cognitive performance throughout almost the entire lifespan among cognitively healthy participants at population level, further studies including cognitively impaired individuals are needed to define cutoff values for CANTAB® or similar computerized neurocognitive test in order to use computerized testing methodology in clinical practice instead of currently used pen and paper test batteries.

This study has several limitations. First, our data are cross-sectional, and repeated assessments of the same individuals using the same neurocognitive instrument are needed to fully study cognitive trajectories. However, the aging effect of the present study is in line with our previous 7-year longitudinal study among the original YFS participants in midlife (G1 generation in the present study) [34], supporting the trajectories obtained in the present study. Second, our test battery did not cover verbal aspects of cognition, and tests focusing on inhibition and delayed recall were also lacking. Third, while the first principal component scores appeared to capture essential characteristics of the dataset, the proportion of explained variance was relatively low, which may have an impact on the results of this study. Another limitation of common principal component analysis is that the numerical results depend on the data, and therefore comparing individual’s cognitive test results to our population-level results is difficult. However, the aim of the study was to study cognitive performance throughout the lifespan as a phenomenon using a single summary score for each cognitive domain rather than reporting multiple different test variables. Finally, the participants of the present study were cognitively healthy, and the results of this study may not reflect general population especially among older populations, in which prevalence of cognitive impairment is common.

To conclude, cognitive performance changes during the lifespan, and multiple factors, such as sex, education, and genetics, modify gains and losses. This unique study of ~ 6500 individuals in three familiarly related generations provides much-needed information about the natural course of cognitive performance from childhood to old age and is thereby an essential reference for further studies aiming at preventing pathological changes in cognition deviating from normal cognitive performance, as well as for studies aiming at promoting cognitive health.

## Supplementary Information

Below is the link to the electronic supplementary material.

Supplementary file1 (DOCX 479 KB)

## Declarations

### Data and materials availability

Cardiovascular Risk in Young Finns Study (YFS) data may be provided by the YFS Steering Group/Data Sharing Committee pending scientific review and a completed material transfer agreement. Requests for the YFS data should be submitted to the PI of the YFS Olli Raitakari (olli.raitakari@utu.fi).
