# Supplementary material for: Cognitive performance from childhood to old age and intergenerational correlations in the multigenerational Young Finns Study
Source: J Neurol. 2024 Sep 22;271(11):7294–308. doi: 10.1007/s00415-024-12693-7 (PMC11561001; doi:10.1007/s00415-024-12693-7)
Supplement: Supplementary file 1 — Supplementary file1 (DOCX 479 KB) [file 415_2024_12693_MOESM1_ESM.docx]

Supplementary Information for

**Cognitive performance from childhood to old age and intergenerational correlations in the multigenerational Young Finns Study**

Marja A. Heiskanen *et al.*

Corresponding author e-mail: [marja.heiskanen@utu.fi](mailto:marja.heiskanen@utu.fi)

**This file includes:**

Online Resources 1-9

**Online Resource** **1** Variable loadings on the three first principal components for overall cognition.

| **Variable** | **First principal component**  Proportion of variance explained  **18.4** | **Second principal component**  Proportion of variance explained  **10.5** | **Third principal component**  Proportion of variance explained  **6.9** |
| --- | --- | --- | --- |
|  | ***Coefficient*** | ***Coefficient*** | ***Coefficient*** |
| PAL – Total attempts (8 patterns) | -0.297 | 0.285 | 0.019 |
| PAL – Total attempts (12 patterns) | -0.293 | 0.251 | 0.023 |
| PAL – Total errors (12 patterns, adjusted) | -0.272 | 0.251 | 0.023 |
| PAL – Total errors (8 patterns, adjusted) | -0.248 | 0.238 | 0.025 |
| PAL – Total attempts (6 patterns) | -0.211 | 0.178 | 0.056 |
| PAL – Total errors (6 patterns, adjusted) | -0.178 | 0.149 | 0.048 |
| PAL – Mean errors to succeed | -0.172 | 0.121 | 0.028 |
| PAL – Total attempts (4 patterns) | -0.102 | 0.036 | 0.038 |
| PAL – Total errors (4 patterns, adjusted) | -0.085 | 0.032 | 0.032 |
| PAL – Total errors (2 patterns, adjusted) | -0.017 | -0.006 | 0.014 |
| PAL – Total attempts (2 patterns) | -0.018 | -0.006 | 0.015 |
| PAL – Number of reached patterns | 0.183 | -0.175 | -0.027 |
| PAL – First time memory score | 0.268 | -0.226 | -0.052 |
| SWM – Strategy (6-8 boxes) | -0.264 | -0.388 | 0.108 |
| SWM – Strategy (6-12 boxes) | -0.268 | -0.370 | 0.066 |
| SWM – Strategy (6 boxes only) | -0.231 | -0.370 | 0.121 |
| SWM – Between errors (8 boxes) | -0.227 | -0.223 | 0.067 |
| SWM – Between errors (6 boxes) | -0.185 | -0.222 | 0.105 |
| SWM – Between errors (12 boxes) | -0.230 | -0.158 | -0.312 |
| SWM – Between errors (4 boxes) | -0.102 | -0.107 | 0.032 |
| SWM – Double errors (12 boxes) | -0.093 | -0.040 | -0.646 |
| SWM – Within errors (8 boxes) | -0.077 | -0.062 | -0.012 |
| SWM – Double errors (8 boxes) | -0.074 | -0.062 | -0.001 |
| SWM – Within errors (12 boxes) | -0.083 | -0.030 | -0.645 |
| SWM – Within errors (6 boxes) | -0.038 | -0.059 | 0.049 |
| SWM – Double errors (6 boxes) | -0.035 | -0.060 | 0.048 |
| SWM – Within errors (4 boxes) | -0.011 | -0.016 | -0.009 |
| SWM – Double errors (4 boxes) | -0.006 | -0.002 | -0.005 |
| RVP – Median response latency | -0.063 | -0.006 | 0.007 |
| RVP – Probability of false alarm | -0.031 | 0.003 | 0.000 |
| RVP – Total false alarm | -0.029 | 0.003 | 0.000 |
| RVP – A’ (sensitivity to target sequence) | 0.183 | 0.049 | 0.008 |
| RVP – Total hits | 0.201 | 0.061 | 0.009 |
| RTI – Error score (inaccurate) | 0.011 | 0.011 | -0.025 |
| RTI – Error score (premature) | -0.017 | -0.002 | -0.004 |
| RTI – Error score (no response) | 0.000 | 0.000 | 0.000 |
| RTI – Median reaction time | -0.067 | -0.013 | -0.039 |
| RTI – Median movement time | -0.056 | 0.002 | 0.003 |

PAL = Paired Associates Learning test; SWM = Spatial Working Memory test; RVP = Rapid Visual Information Processing test; RTI = Reaction Time test.

**Online Resource 2** Domain-specific variable loadings on the three first principal components.

| **Variable** | **First principal component** | **Second principal component** | **Third principal component** |
| --- | --- | --- | --- |
| **Paired Associates Learning test** | Proportion of variance explained  **45.8** | Proportion of variance explained  **15.0** | Proportion of variance explained  **10.1** |
|  | ***Coefficient*** | ***Coefficient*** | ***Coefficient*** |
| PAL – Total attempts (8 patterns) | -0.418 | 0.119 | 0.004 |
| PAL – Total attempts (12 patterns) | -0.402 | 0.244 | -0.008 |
| PAL – Total errors (12 patterns, adjusted) | -0.383 | 0.278 | -0.013 |
| PAL – Total errors (8 patterns, adjusted) | -0.341 | 0.167 | 0.005 |
| PAL – Total attempts (6 patterns) | -0.286 | -0.265 | 0.051 |
| PAL – Total errors (6 patterns, adjusted) | -0.235 | -0.117 | 0.033 |
| PAL – Mean errors to succeed | -0.225 | -0.737 | -0.056 |
| PAL – Total attempts (4 patterns) | -0.114 | -0.209 | 0.124 |
| PAL – Total errors (4 patterns, adjusted) | -0.095 | -0.164 | 0.085 |
| PAL – Total errors (2 patterns, adjusted) | -0.020 | -0.013 | -0.701 |
| PAL – Total attempts (2 patterns) | -0.019 | -0.012 | -0.692 |
| PAL – Number of reached patterns | 0.248 | -0.286 | -0.005 |
| PAL – First time memory score | 0.359 | 0.202 | 0.004 |
| **Spatial Working Memory test** | Proportion of variance explained  **33.3** | Proportion of variance explained  **17.4** | Proportion of variance explained  **11.7** |
|  | ***Coefficient*** | ***Coefficient*** | ***Coefficient*** |
| SWM – Strategy (6-8 boxes) | -0.479 | 0.121 | 0.111 |
| SWM – Strategy (6-12 boxes) | -0.473 | 0.077 | 0.128 |
| SWM – Strategy (6 boxes only) | -0.439 | 0.128 | 0.134 |
| SWM – Between errors (8 boxes) | -0.331 | 0.071 | -0.225 |
| SWM – Between errors (6 boxes) | -0.300 | 0.100 | 0.019 |
| SWM – Between errors (12 boxes) | -0.298 | -0.320 | 0.108 |
| SWM – Between errors (4 boxes) | -0.154 | 0.027 | 0.044 |
| SWM – Double errors (12 boxes) | -0.097 | -0.645 | -0.003 |
| SWM – Within errors (8 boxes) | -0.091 | -0.010 | -0.643 |
| SWM – Double errors (8 boxes) | -0.088 | -0.001 | -0.624 |
| SWM – Within errors (12 boxes) | -0.085 | -0.651 | -0.024 |
| SWM – Within errors (6 boxes) | -0.066 | 0.049 | -0.208 |
| SWM – Double errors (6 boxes) | -0.063 | 0.047 | -0.190 |
| SWM – Within errors (4 boxes) | -0.016 | -0.014 | -0.055 |
| SWM – Double errors (4 boxes) | -0.006 | -0.010 | -0.053 |
| **Rapid Visual Information Processing test** | Proportion of variance explained  **46.8** | Proportion of variance explained  **38.3** | Proportion of variance explained  **14.3** |
|  | ***Coefficient*** | ***Coefficient*** | ***Coefficient*** |
| RVP – Median response latency | -0.208 | -0.032 | -0.977 |
| RVP – Probability of false alarm | -0.055 | -0.684 | 0.034 |
| RVP – Total false alarm | -0.048 | -0.684 | 0.037 |
| RVP – A’ (sensitivity to target sequence) | 0.643 | 0.128 | -0.131 |
| RVP – Total hits | 0.733 | -0.219 | -0.158 |
| **Reaction Time test** | Proportion of variance explained  **32.8** | Proportion of variance explained  **24.9** | Proportion of variance explained  **16.2** |
|  | ***Coefficient*** | ***Coefficient*** | ***Coefficient*** |
| RTI – Error score (inaccurate) | -0.976 | -0.168 | 0.066 |
| RTI – Error score (premature) | -0.051 | -0.058 | -0.995 |
| RTI – Error score (no response) | -0.009 | -0.051 | -0.038 |
| RTI – Median reaction time | 0.071 | -0.809 | 0.021 |
| RTI – Median movement time | 0.197 | -0.558 | 0.056 |

PAL = Paired Associates Learning test; SWM = Spatial Working Memory test; RVP = Rapid Visual Information Processing test; RTI = Reaction Time test.

**Online Resource 3** Second and third principal components of cognitive performance trajectories between ages 7 and 92 years.

**
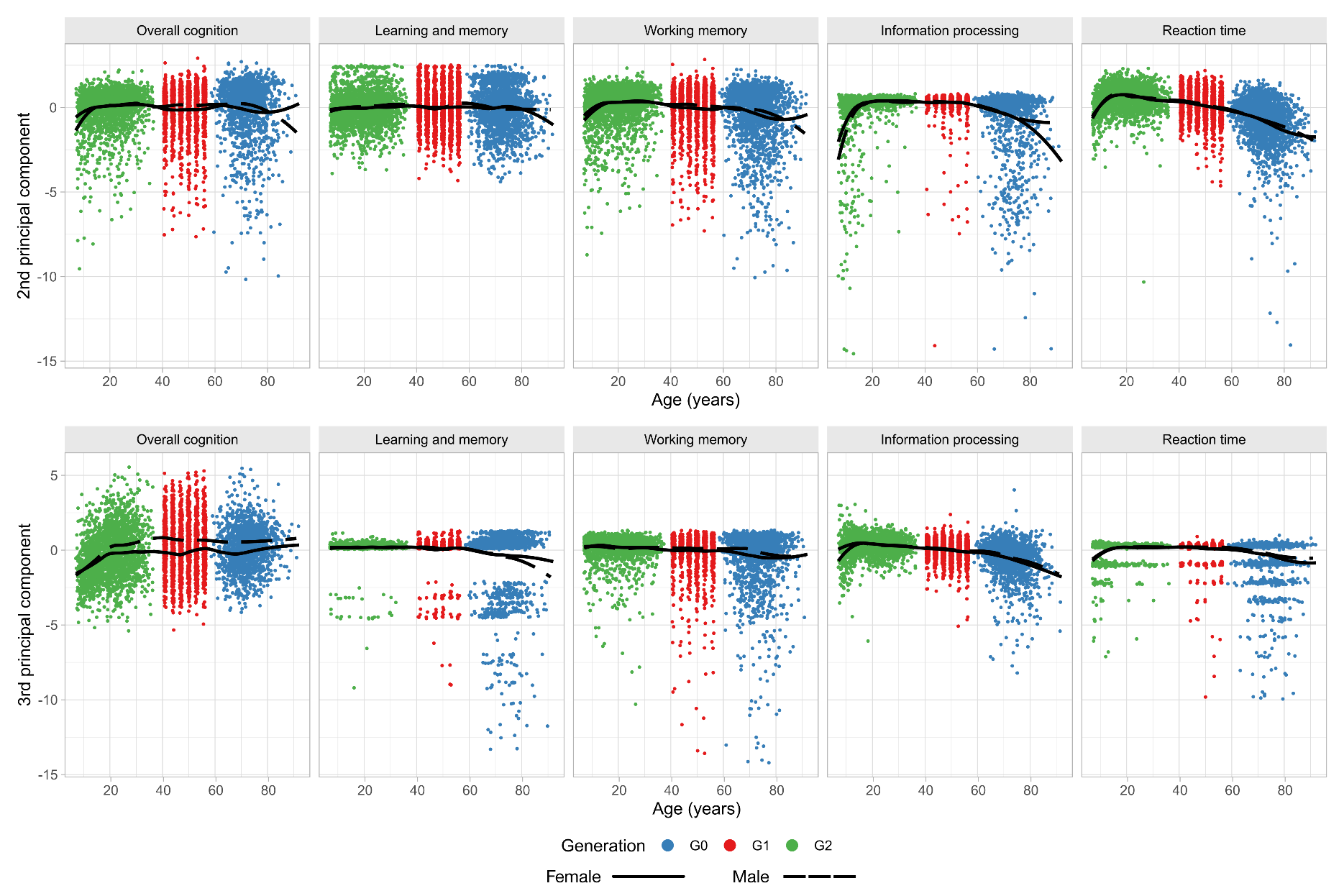
**

The data points represent the second (upper panel) and third principal component scores (lower panel) for each cognitive domain, colored by the generation according to the legend. The trajectories for females and males were obtained by Loess smoothing.

**Online Resource 4** Linear associations of age, sex and education with 1/x transformed reaction time within the generations. The 1/x transformed first principal component scores for reaction time have been standardized in relation to age group 20-29 years (20≤ age <30). Hence, the β estimates describe how many standard deviations (95% confidence interval) the given principal component differs from the highest values reached in age 20-29 years.

|  | **Age, years**  β estimate (95% CI) | **Female sex**  β estimate (95% CI) | **Education, years**  β estimate (95% CI) |
| --- | --- | --- | --- |
| **G0 (59-92 years)** | **-0.01 (-0.02, -0.01**) | 0.05 (-0.03, 0.13) | -0.00 (-0.01, 0.01) |
| **G1 (41-56 years)** | -0.01 (-0.02, 0.00) | 0.03 (-0.07, 0.13) | 0.01 (-0.01, 0.02) |
| **G2 (7-37 years)** | **0.03 (0.01, 0.04)** | **0.32 (0.11, 0.53)** | -0.01 (-0.04, 0.02) |
|  |  |  |  |
| **G2 (25-37 years)** | 0.02 (-0.05, 0.10) | 0.28 (-0.16, 0.71) | -0.02 (-0.10, 0.06) |
| **G2 (18-24 years)** | 0.09 (-0.04, 0.23) | **0.55 (0.02, 1.07)** | -0.02 (-0.11, 0.07) |
| **G2 (13-17 years)** | 0.04 (-0.02, 0.11) | **0.33 (0.15, 0.51)** | -0.00 (-0.03, 0.02) |
| **G2 (7-12 years)** | **0.05 (0.00, 0.10)** | 0.05 (-0.11, 0.22) | -0.02 (-0.04, 0.01) |

**Online Resource 5** Linear associations of age, sex and education with the second principal component scores of reaction time within the generations. The second principal component scores for reaction time have been standardized in relation to age group 20-29 years (20≤ age <30). Hence, the β estimates describe how many standard deviations (95% confidence interval) the given principal component differs from the highest values reached in age 20-29 years.

|  | **Age, years**  β estimate (95% CI) | **Female sex**  β estimate (95% CI) | **Education, years**  β estimate (95% CI) |
| --- | --- | --- | --- |
| **G0 (59-92 years)**  **G1 (41-56 years)**  **G2 (7-37 years)** | **-0.08 (-0.10, -0.07)**  **-0.04 (-0.05, -0 03)**  **0.02 (0.02, 0.03)** | -0.13 (-0.30, 0.05)  **-0.13 (-0.23, -0.03)**  -0.03 (-0.11, 0.06) | 0.02 (-0.01, 0.04)  -0.03 (-0.11, 0.06)  -0.00 (-0.02, 0.01) |
|  | | | |
| **G2 (25-37 years)**  **G2 (18-24 years)**  **G2 (13-17 years)**  **G2 (7-12 years)** | **-0.04 (-0.06, -0.01)**  -0.03 (-0.06, 0.01)  **0.07 (0.02, 0.13)**  **0.34 (0.28, 0.40)** | -0.05 (-0.20, 0.10)  0.07 (-0.06, 0.21)  -0.13 (-0.29, 0.03)  0.05 (-0.16, 0.25) | -0.00 (-0.03, 0.03)  0.01 (-0.01, 0.04)  0.01 (-0.02, 0.03)  0.02 (-0.00, 0.05) |

**Online Resource 6** Age-adjusted correlations between generations G0 and G1.

| **Family relation** | **Cognitive**  **domain** | **r** | **p value** | **n** |
| --- | --- | --- | --- | --- |
| Grandmother-offspring | Overall | 0.20 | **<0.001** | 581 |
| Grandmother-mother | Overall | 0.19 | **0.001** | 314 |
| Grandmother-father | Overall | 0.21 | **0.001** | 267 |
| Grandfather-offspring | Overall | 0.20 | **<0.001** | 440 |
| Grandfather-mother | Overall | 0.19 | **0.005** | 232 |
| Grandfather-father | Overall | 0.24 | **0.001** | 208 |
| Grandmother-offspring | PAL | 0.19 | **<0.001** | 833 |
| Grandmother-mother | PAL | 0.21 | **<0.001** | 456 |
| Grandmother-father | PAL | 0.17 | **0.001** | 377 |
| Grandfather-offspring | PAL | 0.21 | **<0.001** | 564 |
| Grandfather-mother | PAL | 0.22 | **<0.001** | 298 |
| Grandfather-father | PAL | 0.19 | **0.001** | 266 |
| Grandmother-offspring | RTI | 0.04 | 0.186 | 883 |
| Grandmother-mother | RTI | -0.01 | 0.808 | 483 |
| Grandmother-father | RTI | 0.10 | **0.043** | 400 |
| Grandfather-offspring | RTI | 0.05 | 0.265 | 591 |
| Grandfather-mother | RTI | 0.04 | 0.467 | 315 |
| Grandfather-father | RTI | 0.05 | 0.428 | 276 |
| Grandmother-offspring | RVP | 0.22 | **<0.001** | 652 |
| Grandmother-mother | RVP | 0.14 | **0.006** | 360 |
| Grandmother-father | RVP | 0.31 | **<0.001** | 292 |
| Grandfather-offspring | RVP | 0.11 | **0.015** | 477 |
| Grandfather-mother | RVP | 0.07 | 0.253 | 251 |
| Grandfather-father | RVP | 0.17 | **0.011** | 226 |
| Grandmother-offspring | SWM | 0.11 | **0.002** | 888 |
| Grandmother-mother | SWM | 0.09 | **0.047** | 489 |
| Grandmother-father | SWM | 0.11 | **0.022** | 399 |
| Grandfather-offspring | SWM | 0.08 | **0.047** | 587 |
| Grandfather-mother | SWM | 0.05 | 0.416 | 313 |
| Grandfather-father | SWM | 0.13 | **0.029** | 274 |

PAL = Paired Associates Learning test; SWM = Spatial Working Memory test; RVP = Rapid Visual Information Processing test; RTI = Reaction Time test. Statistically significant *p* values are bolded.

**Online Resource 7** Age-adjusted correlations between generations G1 and G2.

| **Family relation** | **Cognitive**  **domain** | **r** | **p value** | **n** |
| --- | --- | --- | --- | --- |
| Mother-offspring | Overall | 0.14 | **<0.001** | 1079 |
| Mother-daughter | Overall | 0.15 | **<0.001** | 631 |
| Mother-son | Overall | 0.14 | **0.004** | 448 |
| Father-offspring | Overall | 0.20 | **<0.001** | 774 |
| Father-daughter | Overall | 0.19 | **<0.001** | 378 |
| Father-son | Overall | 0.20 | **<0.001** | 396 |
| Mother-offspring | PAL | 0.15 | **<0.001** | 1190 |
| Mother-daughter | PAL | 0.14 | **<0.001** | 694 |
| Mother-son | PAL | 0.16 | **<0.001** | 496 |
| Father-offspring | PAL | 0.15 | **<0.001** | 859 |
| Father-daughter | PAL | 0.09 | 0.055 | 418 |
| Father-son | PAL | 0.20 | **<0.001** | 441 |
| Mother-offspring | RTI | 0.08 | **0.008** | 1252 |
| Mother-daughter | RTI | 0.06 | 0.110 | 723 |
| Mother-son | RTI | 0.07 | 0.102 | 529 |
| Father-offspring | RTI | 0.07 | 0.055 | 868 |
| Father-daughter | RTI | 0.05 | 0.298 | 424 |
| Father-son | RTI | 0.11 | **0.026** | 444 |
| Mother-offspring | RVP | 0.22 | **<0.001** | 1129 |
| Mother-daughter | RVP | 0.23 | **<0.001** | 655 |
| Mother-son | RVP | 0.19 | **<0.001** | 474 |
| Father-offspring | RVP | 0.25 | **<0.001** | 792 |
| Father-daughter | RVP | 0.28 | **<0.001** | 389 |
| Father-son | RVP | 0.22 | **<0.001** | 403 |
| Mother-offspring | SWM | 0.13 | **<0.001** | 1261 |
| Mother-daughter | SWM | 0.13 | **0.001** | 731 |
| Mother-son | SWM | 0.13 | **0.002** | 530 |
| Father-offspring | SWM | 0.17 | **<0.001** | 875 |
| Father-daughter | SWM | 0.18 | **<0.001** | 426 |
| Father-son | SWM | 0.18 | **<0.001** | 449 |

PAL = Paired Associates Learning test; SWM = Spatial Working Memory test; RVP = Rapid Visual Information Processing test; RTI = Reaction Time test. Statistically significant *p* values are bolded.

**Online Resource 8** Age-adjusted correlations between generations G0 and G2.

| **Family relation** | **Cognitive**  **domain** | **r** | **p value** | **n** |
| --- | --- | --- | --- | --- |
| Grandmother-grandchild | Overall | 0.04 | 0.267 | 670 |
| Grandmother-granddaughter | Overall | 0.00 | 0.933 | 371 |
| Grandmother-grandson | Overall | 0.09 | 0.112 | 299 |
| Grandfather-grandchild | Overall | 0.10 | 0.051 | 417 |
| Grandfather-granddaughter | Overall | 0.07 | 0.305 | 223 |
| Grandfather-grandson | Overall | 0.11 | 0.119 | 194 |
| Grandmother-grandchild | PAL | 0.06 | 0.066 | 998 |
| Grandmother-granddaughter | PAL | 0.01 | 0.748 | 544 |
| Grandmother-grandson | PAL | 0.11 | **0.020** | 454 |
| Grandfather-grandchild | PAL | 0.09 | **0.031** | 535 |
| Grandfather-granddaughter | PAL | 0.03 | 0.575 | 292 |
| Grandfather-grandson | PAL | 0.17 | **0.009** | 243 |
| Grandmother-grandchild | RTI | -0.05 | 0.134 | 1025 |
| Grandmother-granddaughter | RTI | 0.00 | 0.946 | 566 |
| Grandmother-grandson | RTI | -0.07 | 0.111 | 459 |
| Grandfather-grandchild | RTI | 0.02 | 0.660 | 564 |
| Grandfather-granddaughter | RTI | -0.02 | 0.694 | 308 |
| Grandfather-grandson | RTI | 0.11 | 0.083 | 256 |
| Grandmother-grandchild | RVP | 0.10 | **0.005** | 735 |
| Grandmother-granddaughter | RVP | 0.09 | 0.065 | 408 |
| Grandmother-grandson | RVP | 0.11 | **0.049** | 327 |
| Grandfather-grandchild | RVP | 0.03 | 0.588 | 450 |
| Grandfather-granddaughter | RVP | 0.01 | 0.823 | 234 |
| Grandfather-grandson | RVP | 0.05 | 0.476 | 216 |
| Grandmother-grandchild | SWM | 0.01 | 0.633 | 1038 |
| Grandmother-granddaughter | SWM | -0.02 | 0.681 | 569 |
| Grandmother-grandson | SWM | 0.05 | 0.316 | 469 |
| Grandfather-grandchild | SWM | 0.00 | 0.977 | 559 |
| Grandfather-granddaughter | SWM | 0.01 | 0.843 | 305 |
| Grandfather-grandson | SWM | -0.02 | 0.693 | 254 |

PAL = Paired Associates Learning test; SWM = Spatial Working Memory test; RVP = Rapid Visual Information Processing test; RTI = Reaction Time test. Statistically significant *p* values are bolded.

**Online Resource 9** Age-adjusted correlations for the second principal component scores of reaction time.

| **Family relation** | **Cognitive**  **domain** | **r** | **p value** | **n** |
| --- | --- | --- | --- | --- |
| **G0 and G1** |  |  |  |  |
| Grandmother-offspring | RTI | 0.05 | 0.148 | 883 |
| Grandmother-mother | RTI | 0.00 | 0.936 | 483 |
| Grandmother-father | RTI | 0.13 | **0.007** | 400 |
| Grandfather-offspring | RTI | 0.08 | 0.063 | 591 |
| Grandfather-mother | RTI | 0.06 | 0.279 | 315 |
| Grandfather-father | RTI | 0.10 | 0.097 | 276 |
| **G1 and G2** |  |  |  |  |
| Mother-offspring | RTI | 0.08 | **0.007** | 1252 |
| Mother-daughter | RTI | 0.08 | **0.028** | 723 |
| Mother-son | RTI | 0.06 | 0.175 | 529 |
| Father-offspring | RTI | 0.10 | **0.005** | 868 |
| Father-daughter | RTI | 0.06 | 0.211 | 424 |
| Father-son | RTI | 0.13 | **0.008** | 444 |
| **G0 and G2** |  |  |  |  |
| Grandmother-grandchild | RTI | 0.02 | 0.430 | 1025 |
| Grandmother-granddaughter | RTI | 0.06 | 0.134 | 566 |
| Grandmother-grandson | RTI | -0.03 | 0.480 | 459 |
| Grandfather-grandchild | RTI | 0.00 | 0.945 | 564 |
| Grandfather-granddaughter | RTI | -0.05 | 0.418 | 308 |
| Grandfather-grandson | RTI | 0.05 | 0.419 | 256 |

RTI = Reaction Time test. Statistically significant *p* values are bolded.
